# Characterization of the DREBA4-Type Transcription Factor (SlDREBA4), Which Contributes to Heat Tolerance in Tomatoes

**DOI:** 10.3389/fpls.2020.554520

**Published:** 2020-09-30

**Authors:** Lianzhen Mao, Minghua Deng, Shurui Jiang, Haishan Zhu, Zhengan Yang, Yanling Yue, Kai Zhao

**Affiliations:** College of Horticulture and Landscape, Yunnan Agricultural University, Kunming, China

**Keywords:** Microtom, dehydration-responsive element binding transcription factor, abiotic stress, transgenic plants, gene function, heat tolerance

## Abstract

Dehydration-responsive element binding (DREB) transcription factors play crucial regulatory roles in abiotic stress. The only DREB transcription factor in tomato (*Solanum lycopersicum*), SlDREBA4 (Accession No. MN197531), which was determined to be a DREBA4 subfamily member, was isolated from cv. Microtom using high-temperature-induced digital gene expression (DGE) profiling technology. The constitutive expression of *SlDREBA4* was detected in different tissues of Microtom plants. In addition to responding to high temperature, *SlDREBA4* was up-regulated after exposure to abscisic acid (ABA), cold, drought and high-salt conditions. Transgenic overexpression and silencing systems revealed that *SlDREBA4* could alter the resistance of transgenic Microtom plants to heat stress by altering the content of osmolytes and stress hormones, and the activities of antioxidant enzymes at the physiologic level. Moreover, *SlDREBA4* regulated the downstream gene expression of many heat shock proteins (Hsp), as well as calcium-binding protein enriched in the pathways of protein processing in endoplasmic reticulum (ko04141) and plant-pathogen interaction (ko04626) at the molecular level. SlDREBA4 also induces the expression of biosynthesis genes in jasmonic acid (JA), salicylic acid (SA), and ethylene (ETH), and specifically binds to the DRE elements (core sequence, A/GCCGAC) of the *Hsp* genes downstream from SlDREBA4. This study provides new genetic resources and rationales for tomato heat-tolerance breeding and the heat-related regulatory mechanisms of DREBs.

## Highlights

*SlDREBA4* alters the resistance of transgenic Microtom plants to heat stress by altering the content of osmolytes and stress hormones, the activities of antioxidant enzymes at the physiologic level, and the downstream gene expression of many heat shock proteins, as well as calcium-binding protein at the molecular level.

## Introduction

Dehydration-responsive element binding (DREB) proteins belong to the plant-specific APETALA2/ethylene-responsive element binding factor (AP2/ERF) family of transcription factors. DREBs can be induced by abiotic stresses, including drought, heat, cold, and high salt levels, and their overexpression in transgenic plants increases resistance to these stresses (Wu et al., 2017; Hu et al., 2018; Huang et al., 2018). Abiotic heavy-metal stress, like those induced by cadmium (Cd) and molybdenum (Mo) exposure, can also increase the transcription levels of DREBs, and DREB overexpression results in increased resistance levels against these metal elements (Charfeddine et al., 2017; Akbudak et al., 2018; Nasir and Fazal, 2018). Most previous DREB studies focused on abiotic stresses and a few studies have shown that DREBs play important roles in biotic stress responses. Salicylic acid (SA), jasmonic acid (JA), ethylene (ETH), and other hormones or pathogens induce the up-regulation of DREBs, which then change the resistance levels to these pathogens by altering the content of SA, JA, and ETH, and the expression of pathogenesis-related (PR) genes (Zhao et al., 2015; Wu et al., 2017; Xiong et al., 2018).

DREB transcription factors in the model plant *Arabidopsis thaliana* are divided into six subfamilies (A1 to A6) based on homology (Sakuma et al., 2002). Subsequent studies have classified DREBs from different plants into these six subfamilies. Each subfamily has similar response patterns and functions. After the first isolation of a DREB transcription factor using the single-hybrid yeast method (Liu et al., 1998), the focus of DREB research has mainly involved subfamilies A1, A2, A5, and A6. The response patterns of DREBA1 subfamilies are mainly correlated with responses to abiotic stresses, such as cold, high salt, drought and heavy metals, and biotic stresses, such as pathogenic bacteria. DREBA1 plays positive regulatory roles in responses to these abiotic and biotic stresses (Liu et al., 1998; Kitashiba et al., 2004; Yang et al., 2011; Wei et al., 2017; Wu et al., 2017). The responses and functions of the DREBA2 subfamily to stress are more diverse than those of the DREBA1 subfamily (Sakuma et al., 2006; Zhao et al., 2013; Wu et al., 2018). DREBA2 subfamily transcription factors require post-translational modifications to activate the downstream functional genes and increase resistance to heat and other stresses (Sakuma et al., 2006; Wu et al., 2018). Recent studies on the DREBA2 subfamily mainly focused on heat stress, and they conferred heat resistance to transgenic plants (Zhao et al., 2013; Qin et al., 2018; Wu et al., 2018). The stress-response mechanisms and functions of most transcription factors in the two subfamilies, DREBA5 and DREBA6, are similar to those of the DREBA1 subfamily (Huang and Liu, 2006; Tsutsui et al., 2009; Hu et al., 2018).

To date, only a few DREBA3 and DREBA4 subfamily genes have been isolated, and their regulatory mechanisms and functions under stress conditions are not yet clear. AtABI4 from *Arabidopsis thaliana*, belonging to the DREBA3 subfamily, is induced by ABA (Finkelstein et al., 1998). Another DREBA3-type transcription factor ZmABI4 from maize has an unknown induction mechanism (Niu et al., 2002). For the DREBA4 subfamily of transcription factors, some progress has been made. The transcription of ZmDBF2 is induced by ABA, drought, and high-salt conditions, and it increases the drought resistance of transgenic plants (Wang et al., 2010). The expression of AtTINY under stress conditions has not been explored (Wilson et al., 1996). DaCBF4 from *Deschampsia antarctica* has been isolated recently. Its transcriptional level increases during cold and drought stress, and it increases the cold resistance of transgenic plants (Byun et al., 2018). ZmDREB4.1 could not be induced by high salt, drought, ABA, auxin, cytokinins, gibberellic acid, or ETH, and it inhibited the growth of transgenic tobacco leaves, hypocotyls, and callus. However, its functions under stress conditions have not been explored (Li et al., 2018).

After the discovery of DRE element and DREB transcription factors (Yamaguchi-Shinozaki and Shinozaki, 1994; Liu et al., 1998), subsequent studies revealed that DREB plays important roles in both abiotic and biotic stress responses. In this study, the only tomato (*Solanum lycopersicum*) DREB transcription factor that responded to heat, SlDREBA4, was discovered in the cultivar Microtom, using DGE profiling technology. SlDREBA4 was determined to belong to the DREBA4 subfamily. The function of SlDREBA4 under high-temperature conditions was verified through transgenic overexpression and silencing systems. The resistance changes caused by SlDREBA4 under heat-stress conditions were explained at physiological and molecular levels.

## Materials and Methods

### Materials and Heat Induction

The seeds of *S. lycopersicum* L. (var. Microtom) were first soaked in water at 55°C for 20 min, then in water at 30°C for 5 h. The treated seeds of *S. lycopersicum* L. (var. Microtom) were sown in a mixed matrix of 6:3:1 (v/v) peat, vermiculite and perlite. For heat treatment, Microtom plants with seven to eight leaves were treated at 37°C for 30 min. Both the control and heat-treated groups contained 30 plants. The 4^th^ leaves from the base of each plant were removed. The 30 leaves of the control (Microtom-CK) and heat-treated (Microtom-HS) groups were mixed to form two independent materials for DGE profiling, which was performed by the Beijing Genomics Institution (BGI).

### Screening of Differentially Expressed *DREB* Genes Induced by Heat

Based on Poisson distribution analysis (Audic and Claverie, 1997), a strict algorithm was used to identify differentially-expressed genes (DEGs) between two samples. The number of unambiguous clean tags from gene A was denoted by an x, given that the expression of every gene occupies only a small part of the library; x yields to the Poisson distribution:

$$p(x)=\frac{e^{-\lambda }\lambda^{x}}{x!}\text{ (}\lambda \text{ is the real transcripts of the gene)}$$

The total clean tag number of sample 1 is designated N1 and the total clean tag number of sample 2 is designated as N2. Gene A holds x tags in sample 1 and y tags in sample 2. The probability of gene A expressed equally between two samples can be calculated as follows:

2∑i=0i=yp(i|x)or 2×(1−∑i=0i=yp(i|x))(if ∑i=0i=yp(i|x)>0.5)p(y|x)=(N2N1)y(x+y)!x!y!(1+N2N1)(x+y+1)

The P-value corresponds to the DGE test. The false discovery rate (FDR) was used to determine the threshold of the P-value in multiple tests (Benjamini and Yekutieli, 2001). DEGs between two samples (control and heat-treated) were screened, and the genes with FDR ≤ 0.001 and fold-change differences > 2 were selected as the candidate genes. A hypergeometric test was used to find the pathways significantly enriched (Q-value ≤ 0.05) with DEGs. The conserved AP2 sequence of the DREB transcription factor was download from PFAM (http://pfam.sanger.ac.uk/). A local BLAST program was used to compare AP2 sequences with the differentially expressed protein sequences to screen for the DREB transcription factor possessing only one AP2 domain (Yuan et al., 2013).

### *SlDREBA4* Cloning and Phylogenetic Tree Construction

The specific primer pSlDREBA4 (**Table S1**) was designed based on the *SlDREBA4 *gene sequence. *SlDREBA4 *was cloned and sequenced using Microtom cDNA as the template. A phylogenetic tree was established by the Neighbor-Joining algorithm with 1,000 bootstrap replications using MEGA5 version 5.05 software (Sakuma et al., 2002).

### Identification of Tissue-Specific Expression and Stress-Response Patterns of *SlDREBA4 *Using Real-Time Quantitative PCR (RT-qPCR)

Microtom seeds were germinated and sown in vermiculite. After growing two true leaves, the plants were transplanted into Hoagland’s nutrient solution. After seven to eight leaves were grown, plants were exposed to stresses. For the dehydration stress, hydroponic seedlings fixed in planting baskets were transferred from nutrient solution along with the basket to the filter paper. For heat and cold stresses, hydroponic seedlings were exposed to 37°C and 4°C, respectively, in a growth chamber. For salt stress, hydroponic seedlings were transferred into Hoagland’s nutrient solution containing 200 mM NaCl. For the ABA treatment, leaves of hydroponic seedlings were sprayed with 200 mM ABA solution. For all the treatments, the fourth leaves from the base were harvested at the designated time intervals (0, 0.5, 1, 3, 6, and 12 h). When the Microtom plants had red fruit approximately 60 days after sowing, tissue-specific expression was identified in the root, stem, leaf, flower, green fruit, and red fruit. The relative quantification value for the *SlDREBA4* (pSlDREBA4-q) gene was calculated using the 2^−ΔΔCt^ method (Zhao et al., 2013) with the tomato housekeeping gene ribosomal protein L2 (*RPL2*) (pRPL2-q) (Løvdal and Lillo, 2009) as an internal control. Primer sequences are provided in **Table S1**. All the above RT-qPCR analyses were performed in biological and technical triplicate.

### Generation of Transgenic Microtom Lines

To generate overexpressed transgenic Microtom plants, full-length *SlDREBA4* cDNA was amplified using the specific primer, pSlDREBA4-o. The PCR product was fused into the reconstructed vector pBI121 (harboring *Xba*I and *Kpn*I sites) under the control of the constitutive CaMV 35S promoter. The pBI121-35S-*SlDREBA4* fusion plasmid was introduced into *Agrobacterium tumefaciens* strain GV3101. Positive GV3101 was transformed into Microtom cotyledons (Sun et al., 2015), and the rooting plants were screened by kanamycin and transferred to Hoagland’s nutrient solution. The level of *SlDREBA4* transcription was assessed using RT-qPCR with the primer, pSlDREBA4-q. To generate virus-induced gene silencing (VIGS) transgenic Microtom plants, a specific primer, pSlDREBA4-v, was designed based on a sequence specificity analysis of *SlDREBA4* with other *DREBs* in tomatoes. The gene fragment was then cloned and inserted into the pTRV2 plasmid. The plasmids, pTRV2-*SlDREBA4* segment, pTRV2-PDS (phytoene desaturase), and pTRV1, were transformed into GV3101. Positive GV3101 containing pTRV2, pTRV2-*SlDREBA4* segment, or pTRV2-PDS were co-injected with positive GV3101 containing pTRV1 into cotyledons and leaves of Microtom (Shen et al., 2014). Plant phenotypes were observed 21 days after injection, and positive transgenic plants were identified using RT-qPCR with the primer, pSlDREBA4-q. Primers are provided in **Table S1**. All the above RT-qPCR analyses were performed in biological and technical triplicate.

### The Heat-Stress Treatment and Determination of Physiologic and Molecular Indices

To achieve the rapid wilting effect and avoid the influence of plant phenotypic difference on high temperature treatment, the heat-treated groups were placed at 55°C in a plant growth chamber until whole wilted plant was observed. Heat-treated samples were immediately divided into two batches: one batch had its physiological indexes measured and underwent transcriptome sequencing. Another batch was transferred to normal conditions for 3 d, followed by phenotypic observation. According to the specific phenotypic changes under high-temperature stress treatment, the heat injury index (HII) grading criteria was established as follows: level 0, no leave curling; level 1, 0% to 50% leave curling with partial leave wilting; level 2, 50% to 100% leave curling with partial leave wilting; and level 3, the entire plant wilted or withered.

$$\begin{array}{l} \text{HII}(\%)=\Sigma \;(\text{plant numbers at each level}\times \text{ level rank})\\ \;\times \;100/(\text{highest level rank}\times \;\text{the total number of plants}) \end{array}$$

The concentrations of indole-3-acetic acid (IAA), ABA, JA, SA, and 1-aminocyclopropane-1-carboxylic acid (ACC) were measured by HPLC-MS/MS using Agilent 1290 HPLC (Agilent Technologies Inc., USA) and SCIEX-6500 Qtrap mass spectrometer (AB Sciex, USA) (Pan et al., 2010). Proline, malondialdehyde, soluble total sugar, soluble starch, peroxidase (POD), superoxide dismutase (SOD) and catalase (CAT) were detected using an ELISA kit (Shanghai Enzyme-linked Biotechnology Co., Ltd). Plasma membrane permeability was measured in terms of electrolyte leakage rate (Kumar et al., 1985). All the physiologic indices were measured in biological and technical triplicate.

Biosynthetic genes in the JA, SA, and ETH pathways were searched against the DEGs data in the *SlDREBA4*-overexpressed and -silenced lines, and the expression profiles of these genes were validated using RT-qPCR by the 2^-ΔΔCt^ method (Zhao et al., 2013). All the primers are provided in **Table S1**.

The *SlDREBA4*-overexpressed and *-*silenced, and empty vector-transformed plants were selected to carry out transcriptome sequencing. Readcount for each gene was normalized to reads per kilobase per million mapped reads (RPKM). Prior to differential gene expression analysis for each sequenced library, the readcounts were adjusted using the edgeR program package through one scaling normalized factor (Robinson et al., 2010). Analyses of significant differences in gene expression were performed using DESeq2 and the R package (Anders and Huber, 2010). Genes with a p_adj_ < 0.05 and |log_2_(foldchange)| >1 were selected as candidate genes. Based on the principles of hypergeometric distribution, clusterProfiler was used to perform a Kyoto Encyclopedia of Genes and Genomes (KEGG) pathway enrichment analysis of DEGs [p_adj_ < 0.05] (Mortazavi et al., 2008).

### DRE-Binding Activities of SlDREBA4 Using the Yeast One-Hybrid (Y1H) System

Three potentially DREs (DRE1 [GACCGACGA], DRE2 [AGCCGACAC], and DRE3 [CGCCGACTT]) identified from the *Hsp* genes downstream from SlDREBA4 or one mutant DRE (mDRE [TATTTTCAT]) were inserted into the pHis2.1 vector with three tandemly repeats using *Eco*RI and *Spe*I to generate pHis2.1-3DRE or pHis2.1-3mDRE. The coding sequence of *SlDREBA4* was amplified by the primer, pSlDREBA4-y, harboring *Bam*HI and *Xho*I sites (**Table S1**). The product was inserted into the pGADT7 vector to generate pGADT7-*SlDREBA4*. The yeast strain, Y187, co-transformed by the corresponding vectors, was streaked on the selective SD medium to investigate the DRE-binding activities of SlDREBA4 (Wu et al., 2018).

## Results

### Selection of DREB Transcription Factors Responding to Heat Using DGE Profiling

**Figure 1:**
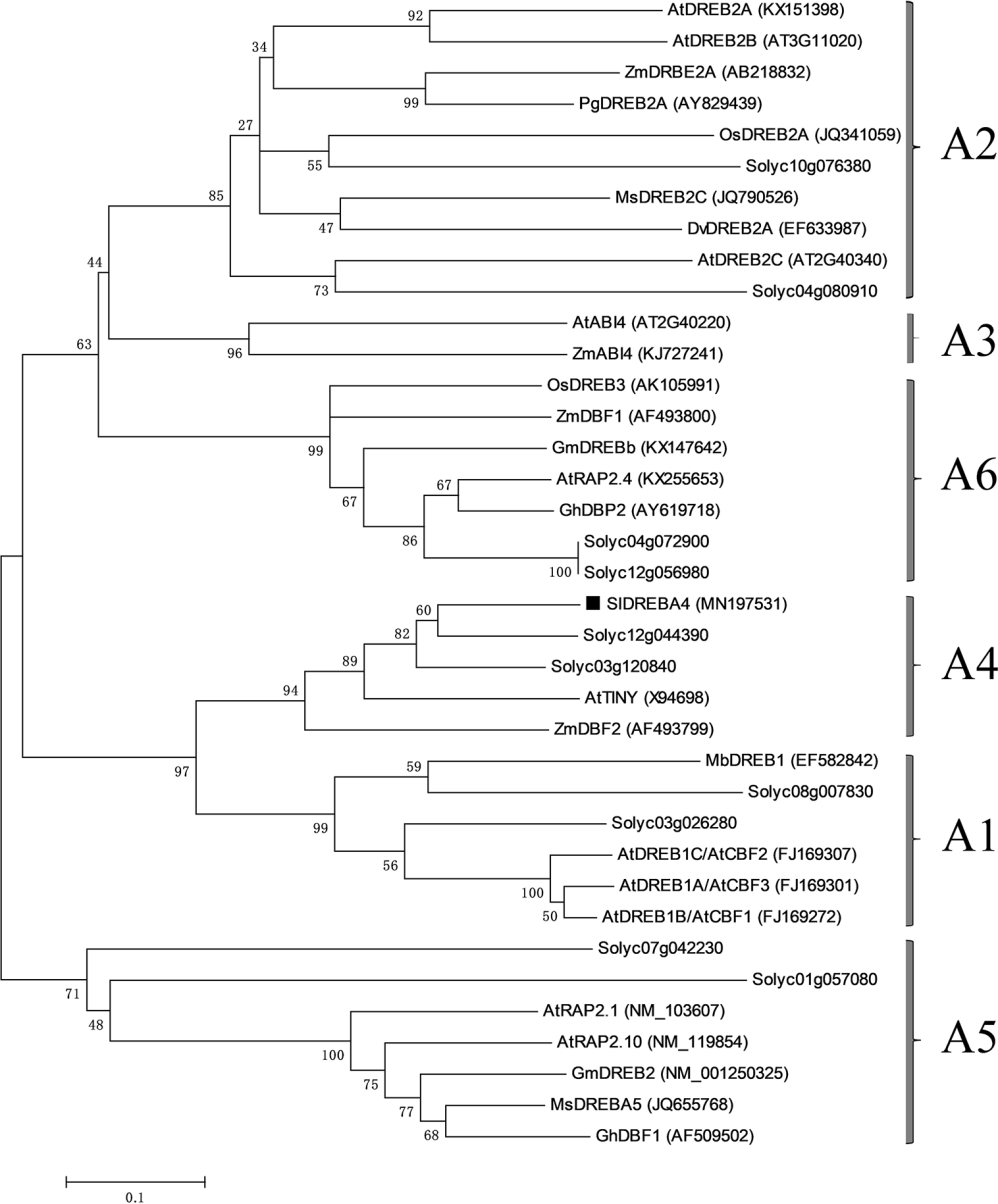
Phylogenetic tree of SlDREBA4 and DREB proteins of other plants. SlDREBA4 is marked with black squares and the accession numbers are indicated in parentheses. The DREB proteins without accession numbers are discovered from tomatoes.

DGE profiling successfully selected 2,820 DEGs responding to heat, including 1,107 up-regulated genes and 1,713 down-regulated genes, which were listed in an excel. Only one DREB subfamily gene (Gene ID: Solyc06g066540) was up-regulated with a log2 ratio=4.28. The coding region contained 579 bp bases and encoded 192 amino acids with one typical AP2 domain. The phylogenetic tree was established, and the gene was found to be a member of the DREBA4 subfamily. Thus, it was named SlDREBA4 (Accession No. MN197531; **Figure 1**).

### Expression Patterns of *SlDREBA4* in Different Tissues and Response to Various Stresses

**Figure 2:**
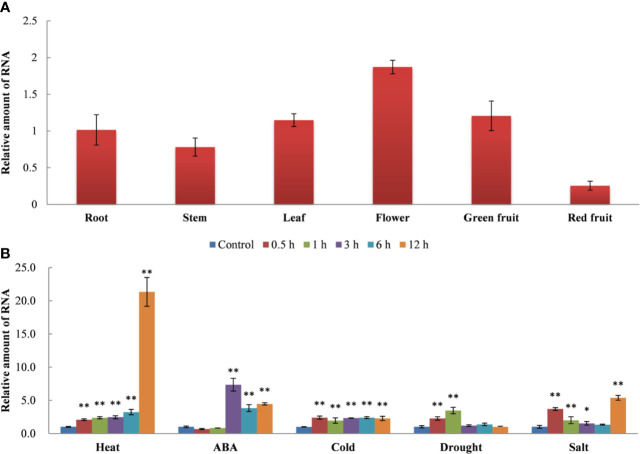
Tissue-specific expression **(A)** and stress-response patterns **(B)** of *SlDREBA4*. The unstressed expression level (Control) was assigned a value of 1. The error bar on each column represents the standard deviation (SD) of the three biological replicates (Student’s *t*-test, *P < 0.05, **P < 0.01).

The expression pattern of *SlDREBA4* in different tissues of Microtom plants was examined under normal conditions (**Figure 2A**). *SlDREBA4* was constitutively expressed in almost all tissues examined, including root, stem, leaf, flower, green fruit, and red fruit. The expression level of *SlDREBA4* was highest in flower, followed by green fruit, leaf, root and stem, and was lowest in red fruit.

We also tested the expression pattern of *SlDREBA4* in the leaves under various stress conditions. RT-qPCR revealed that *SlDREBA4* expression was induced by heat, exogenous ABA, cold, drought and salt (**Figure 2B**). Under different stress conditions, *SlDREBA4* showed different expression patterns. *SlDREBA4* expression was rapidly and transiently induced by the heat treatment and peaked at 12 h. ABA and drought resulted in the increased, followed by decreased expression of *SlDREBA4*. The transcription level of *SlDREBA4* peaked at 3 h after the ABA treatment and then declined, but the expression level at 12 h was still higher than that of the controls. Drought stress resulted in the expression level of *SlDREBA4* peaking at 1 h, followed by a decline, and then it maintained at the same level as the controls. Cold led to the expression level of *SlDREBA4* peaking at 0.5 h, and then, it maintained this expression level until 12 h. Salinity caused an increase in the expression of *SlDREBA4*, followed by a decrease and another increase, with an eventual peak at 12 h.

### Functional Verification of *SlDREBA4* Transgenic Microtom Plants Under Heat Stress Conditions

**Figure 3:**
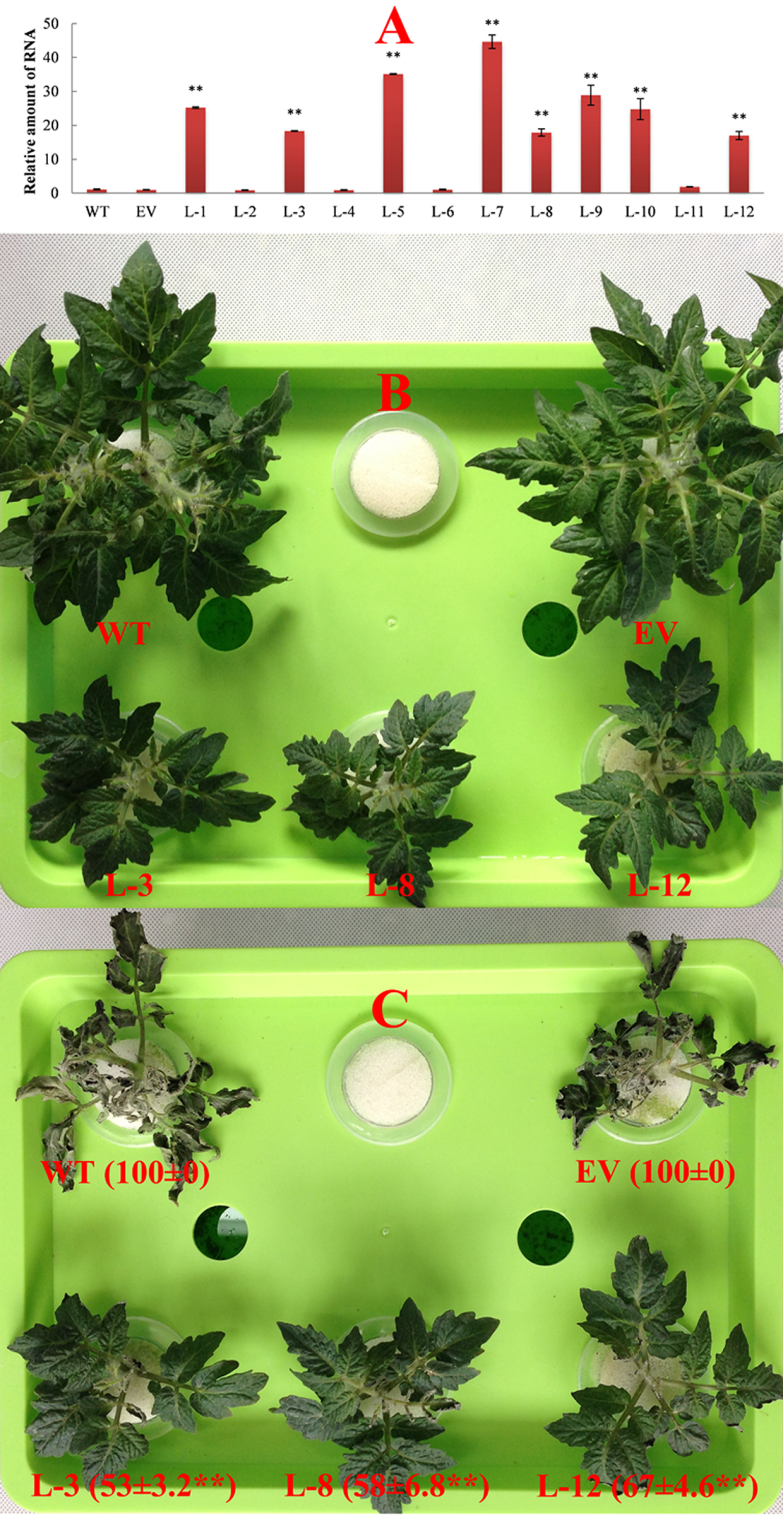
Functional identification of *SlDREBA4-*overexpressing lines under heat-stress conditions. WT, wild-type plants; EV, empty vector-transformed plants; L-1 to L-12: *SlDREBA4*-overexpression lines. **(A)** RT-qPCR was used to identify *SlDREBA4* expression. The level of WT was assigned a value of 1. The error bar on each column represents the SD of the three biological replicates (Student’s *t*-test, ^**^P < 0.01). Plants under normal **(B)** and heat-stress **(C)** conditions were grown in nutrient solution. The numbers in brackets indicate the HII values (%), which are shown as the means of the three biological replicates ± SDs (Student’s *t*-test, ^**^P < 0.01).

Expression of the transgenic three *SlDREBA4*-overexpression lines (L-3, L-8, and L-12) were obtained using RT-qPCR (**Figure 3A**). Compared with the wild-type and empty vector-transformed plants, *SlDREBA4*-overexpression lines were significantly dwarfed, with slow growth and delayed flowering (**Figure 3B**). Heat stress led to wilted wild-type and empty vector-transformed tomato plants with HIIs of 100%. However, only a small portion of leaves on the three transgenic lines were slightly wilted, with HIIs ranging from 53% to 67% (**Figure 3C**).

**Figure 4:**
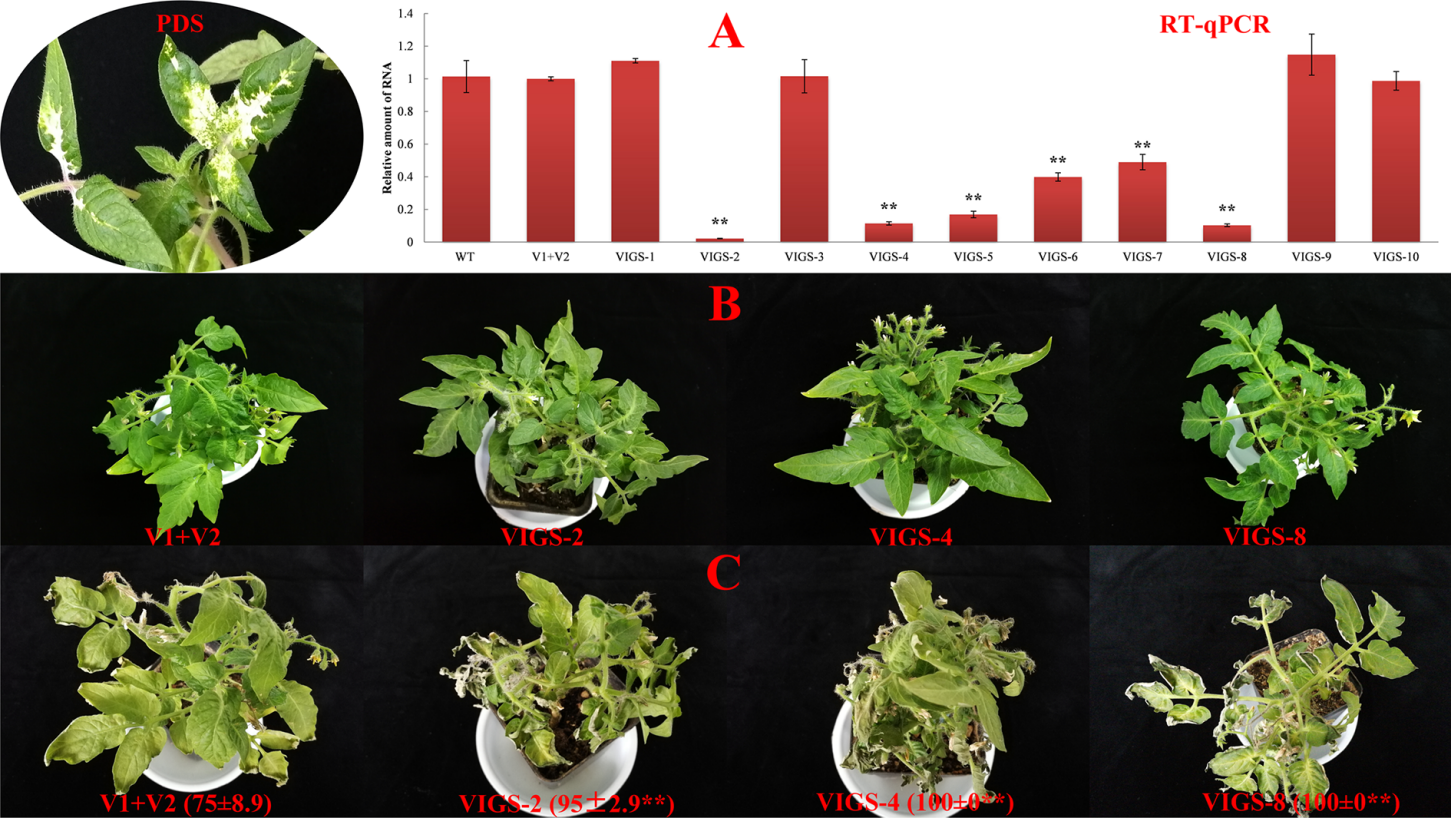
Functional identification of *SlDREBA4-*silenced plants under heat-stress conditions. WT, wild-type plants; V1+V2: plants co-transformed by empty vectors pTRV1 and pTRV2; VIGS-1 to -10: *SlDREBA4-*silenced plants. **(A)** Identification of *SlDREBA4-*silenced plants, PDS, phenotypes of the plants with silenced *PDS*, RT-qPCR: transcription level of *SlDREBA4* using RT-qPCR in which the level of WT was assigned a value of 1. Error bar on each column represents the SD of the three biological replicates (Student’s *t*-test, **P < 0.01). Silenced plants under normal **(B)** and heat-stress **(C)** conditions were grown in nutrient solution. The numbers in brackets indicate the HII values (%), which are shown as the means of the three biological replicates ± SDs (Student’s *t*-test, **P < 0.01).

The function of *SlDREBA4* under heat-stress conditions was determined using the VIGS system. Positive transgenic plants were obtained through the phenotypic analysis of PDS silencing and the expression level of SlDREBA4, and shown under normal conditions (**Figures 4A, B**). After the heat-stress treatment, leaves of *SlDREBA4*-silenced lines were more seriously damaged compared with empty vectors-transformed plants. The HII of empty vectors-transformed plants was 75%, while the HIIs of *SlDREBA4*-silenced lines ranged from 95% to 100% (**Figure 4C**).

### The Causes for the Heat Tolerance Changes of *SlDREBA4*-Transgenic Lines

The causes of changes in the heat tolerance of transgenic lines were investigated at the physiological and molecular levels. The hormone content, enzyme activity, metabolite level, and gene expression level were measured, also. The contents of JA, SA, ACC, soluble total sugar, soluble starch, and proline, and the activities of POD, SOD and CAT in overexpressed lines under normal conditions were found to be higher than those of empty vector-transformed lines. Increased contents and activities of the above substances were measured under heat-stress conditions and were higher in overexpressed lines than in empty vector-transformed lines. Heat induction led to an increase in the ABA level, but no significant difference was observed between the level in empty vector-transformed and overexpressed lines under normal and heat-stress conditions. Under normal growth conditions, there were no significant differences in malondialdehyde level and electrolyte leakage rate between empty vector-transformed and overexpressed lines. Both indexes increased under heat-stress conditions, and increases in the indexes of overexpressed plants were significantly lower than those of empty vector-transformed lines. Heat decreased the IAA level, and under both normal and heat-stress conditions, the IAA level in overexpressed lines was extremely significantly lower than in empty vector-transformed lines (**Table 1**).

**Table 1:** Physiologic indicators in *SlDREBA4*-overexpression and empty vector-transformed lines.

	Control				Heat stress			
	EV	L-3	L-8	L-12	EV	L-3	L-8	L-12
Proline (ng/g·FW)	85.2 ± 6.2	132.8 ± 11.9^**^	142.3 ± 13.9^**^	156.6 ± 15.8^**^	178.8 ± 10.5	458.6 ± 40.5^**^	487.8 ± 25.9^**^	603.6 ± 36.8^**^
Malondialdehyde (nmol/g·FW)	83.9 ± 7.2	76.9 ± 6.4	80.5 ± 7.8	75.9 ± 8.6	344.9 ± 23.6	189.3 ± 15.3^**^	145.9 ± 16.9^**^	159.8 ± 18.9^**^
Soluble total sugar (mg/g·FW)	21.4 ± 1.5	39.6 ± 2.1^**^	30.9 ± 1.8^**^	38.81 ± 1.0^**^	42.8 ± 5.2	78.9 ± 6.3^**^	91.6 ± 4.6^**^	82.3 ± 3.6^**^
Soluble starch (mg/g·FW)	21.7 ± 1.3	26.3 ± 2.3^*^	29.6 ± 3.1^*^	28.7 ± 1.5^*^	45.6 ± 1.2	82.6 ± 5.3^**^	75.1 ± 3.6^**^	91.2 ± 2.9^**^
Electrolyte leakage rate (%)	16.8 ± 0.9	15.6 ± 1.6	16.7 ± 0.8	15.4 ± 1.2	82.8 ± 9.6	36.1 ± 4.3^**^	35.4 ± 4.1^**^	38.9 ± 1.6^**^
POD (U/g·FW)	1.8 ± 0.1	5.7 ± 0.1^**^	6.6 ± 0.2^**^	4.6 ± 0.1^**^	5.4 ± 0.1	20.1 ± 0.5^**^	25.8 ± 0.3^**^	30.8 ± 0.4^**^
SOD (U/g·FW)	1654.6 ± 78.9	3335.8 ± 121.8^**^	3708.6 ± 198.6^**^	3658.3 ± 154.6^**^	2269.6 ± 45.5	6366.5 ± 69.5^**^	6432.5 ± 157.6^**^	8239.6 ± 110.6^**^
CAT (U/g·FW)	504.4 ± 56.4	935.1 ± 35.9^**^	919.2 ± 23.6^**^	990.9 ± 32.5^**^	909.8 ± 90.6	2023.9 ± 58.6^**^	2056.8 ± 115.6^**^	2123.8 ± 69.3^**^
ABA (ng/g·FW)	44.7 ± 1.5	49.2 ± 5.8	48.9 ± 6.7	48.1 ± 5.2	127.9 ± 5.9	123.8 ± 9.1	117.7 ± 6.8	122.8 ± 8.3
JA (ng/g·FW)	17.2 ± 2.1	81.3 ± 7.1^**^	91.6 ± 3.9^**^	87.5 ± 9.5^**^	38.8 ± 2.3	245.6 ± 22.6^**^	278.6 ± 30.2^**^	215.9 ± 11.9^**^
SA (ng/g·FW)	5.5 ± 0.2	39.7 ± 3.6^**^	45.3 ± 6.4^**^	36.8 ± 1.5^**^	10.6 ± 0.9	156.8 ± 14.5^**^	145.6 ± 12.6^**^	158.5 ± 10.3^**^
ACC (ng/g·FW)	756.6 ± 56.9	2063.8 ± 235.6^**^	2653.8 ± 214.6^**^	2145.8 ± 98.6^**^	1231.5 ± 156.3	3684.1 ± 312.5^**^	4123.8 ± 396.5^**^	3569.8 ± 212.3^**^
IAA (ng/g·FW)	0.79 ± 0.06	0.2 ± 0.01^**^	0.16 ± 0.01^**^	0.18 ± 0.01^**^	0.46 ± 0.05	0.1 ± 0.01^**^	0.13 ± 0.01^**^	0.15 ± 0.01^**^

EV, empty vector-transformed lines; L-3, L-8, and L-12, SlDREBA4-overexpression lines. The results are indicated as the means of the three biological replicates ± SDs (Student’s t-test, *P < 0.05, **P < 0.01).

The contents of proline, malondialdehyde, soluble total sugar, soluble starch, ABA, JA, SA, and ACC, and the values of electrolyte leakage rate, and the activities of POD, SOD, and CAT were induced and the content of IAA was suppressed under heat-stress conditions in both empty vector-transformed and *SlDREBA4*-silenced plants. Under both normal growth and heat conditions, physiologic indicators, including proline, POD, SOD, JA, SA, and ACC, were lower in the silenced plants than in the empty vector-transformed plants, whereas the other indicators, including malondialdehyde, soluble total sugar, soluble starch, CAT, ABA, and IAA, showed no significant difference in both silenced and empty vector-transformed plants. Under normal growth conditions, there was no significant difference in the electrolyte leakage rate between empty vector-transformed and silenced plants; however, the electrolyte leakage rate was higher in silenced plants than in empty vector-transformed plants under heat-stress conditions (**Table 2**).

**Table 2:** Physiological indicators in *SlDREBA4*-silenced plants.

	Control				Heat stress			
	V1+V2	VIGS-2	VIGS-4	VIGS-8	V1+V2	VIGS-2	VIGS-4	VIGS-8
Proline (ng/g·FW)	79.8 ± 3.3	69.3 ± 5.0^*^	64.2 ± 7.5^*^	65.4 ± 5.6^*^	167.5 ± 13.8	120.8 ± 8.9^**^	113.7 ± 9.0^**^	119.8 ± 8.8^**^
Malondialdehyde (nmol/g·FW)	78.7 ± 6.9	76.0 ± 5.6	81.6 ± 6.3	79.0 ± 6.9	289.8 ± 12.9	303.3 ± 23.8	295.8 ± 20.1	318.3 ± 30.8
Soluble total sugar (mg/g·FW)	19.0 ± 0.9	19.4 ± 1.2	20.6 ± 2.0	18.9 ± 1.6	36.9 ± 4.1	37.0 ± 4.2	32.9 ± 1.4	31.9 ± 2.7
Soluble starch (mg/g·FW)	32.1 ± 2.9	35.8 ± 2.0	31.5 ± 2.9	36.0 ± 4.4	59.3 ± 4.3	55.9 ± 4.6	56.0 ± 6.5	50.9 ± 5.8
Electrolyte leakage rate (%)	23.9 ± 1.8	24.5 ± 0.9	21.8 ± 2.1	24.5 ± 3.9	68.0 ± 3.1	81.0 ± 6.6^*^	79.0 ± 9.2^*^	76.8 ± 7.9^*^
POD (U/g·FW)	2.3 ± 1.2	1.6 ± 0.1^*^	1.6 ± 0.1^*^	1.8 ± 0.02^*^	7.8 ± 4.5	5.2 ± 0.4^**^	4.8 ± 0.1^**^	5.1 ± 0.5^**^
SOD (U/g·FW)	1465.6 ± 67.8	1254.8 ± 89.7^*^	1189.7 ± 123.7^*^	1134.7 ± 134.7^*^	3078.9 ± 445.8	2078.4 ± 312.7^**^	2134.5 ± 178.9^**^	2218.5 ± 221.7^**^
CAT (U/g·FW)	476.8 ± 34.5	465.7 ± 34.6	509.4 ± 56.7	489.7 ± 34.6	789.9 ± 87.3	800.3 ± 39.7	768.7 ± 56.7	813.8 ± 70.4
ABA (ng/g·FW)	35.8 ± 4.3	38.6 ± 2.9	40.1 ± 4.2	36.8 ± 4.1	81.4 ± 4.0	76.5 ± 4.3	83.6 ± 7.9	74.5 ± 6.9
JA (ng/g·FW)	21.9 ± 1.5	8.8 ± 0.5^**^	7.3 ± 0.9^**^	8.3 ± 0.6^**^	58.9 ± 4.9	25.3 ± 1.3^**^	25.6 ± 1.6^**^	24.6 ± 1.9^**^
SA (ng/g·FW)	9.7 ± 0.2	6.6 ± 0.6^**^	5.9 ± 0.6^**^	4.9 ± 0.3^**^	28.9 ± 1.3	10.6 ± 1.3^**^	11.8 ± 1.2^**^	8.4 ± 0.7^**^
ACC (ng/g·FW)	1091.0 ± 114.3	265.2 ± 23.2^**^	273.6 ± 25.6^**^	337.5 ± 28.3^**^	2584.2 ± 22.3	568.2 ± 33.6^**^	568.5 ± 69.6^**^	756.3 ± 53.6^**^
IAA (ng/g·FW)	0.69 ± 0.05	0.73 ± 0.04	0.71 ± 0.07	0.71 ± 0.04	0.47 ± 0.03	0.51 ± 0.05	0.53 ± 0.0	0.48 ± 0.04

V1+V2: plants co-transformed by empty vectors pTRV1 and pTRV2, VIGS-2, VIGS-4, and VIGS-8: SlDREBA4-silenced plants. The results are indicated as the means of the three biological replicates ± SDs (Student’s t-test, *P < 0.05, **P < 0.01).

**Figure 5:**
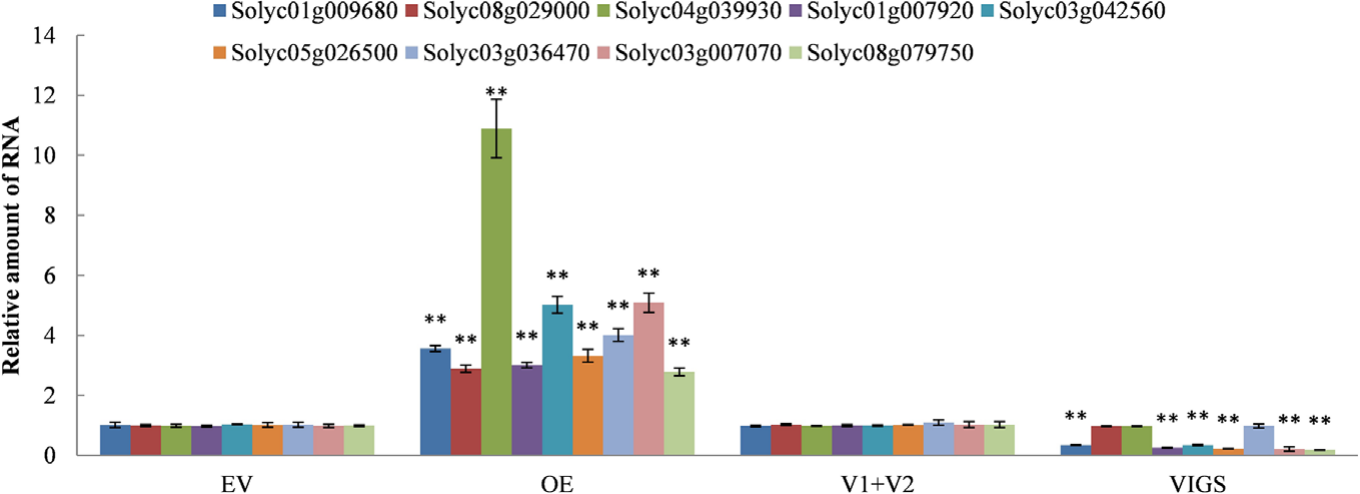
Expression patterns of the DEGs in the JA, SA, and ETH biosynthesis pathways. The expression of the EV and V1+V2 plants were assigned a value of 1. The error bar on each column represents the SD of the three biological replicates (Student’s *t*-test, **P < 0.01).

We continued to dig for the target DEGs in the JA, SA, and ETH biosynthesis pathways against the DEGs data in the transcriptome of *SlDREBA4*-overexpressed and -silenced lines. The results showed that the target DEGs were distributed in each hormone biosynthesis pathway. Three, four, and two DEGs were found in JA, SA, and ETH biosynthesis, respectively. All the DEGs were induced in the *SlDREBA4*-overexpressed lines. However, one (JA), three (SA), and two (ETH) DEGs were inhibited and the remaining genes were not detected in the silenced plants (**Table 3**). RT-qPCR was employed to confirm the differential expression patterns of these biosynthesis genes in the leaves. The expression trends of the lipoxygenase and 12-oxophytodienoate reductase-like protein genes in JA biosynthesis, isochorismatase, and phenylalanine ammonia-lyase genes in SA biosynthesis, and 1-aminocyclopropane-1-carboxylate synthase gene in ETH biosynthesis were in agreement with the transcript abundance changes (**Figure 5**).

**Table 3:** DEGs in the JA, SA, and ETH biosynthesis pathways in the *SlDREBA4*-overexpressed and silenced plants.

Gene ID	Readcount (OE/EV)	Readcount [VIGS/(V1+V2)]	log_2_FoldChange [(OE/EV)/(VIGS/V1+V2)]	Definition	Hormone biosynthetic pathway
Solyc01g009680	259.96/121.66	209.90/458.45	1.10/−1.12	Lipoxygenase	JA
Solyc08g029000	199.00/130.26	—	0.60	Lipoxygenase	JA
Solyc04g039930	8.18/1.67	—	2.32	12-oxophytodienoate reductase-like protein	JA
Solyc01g007920	383.01/252.28	223.35/332.24	0.60/−0.57	Isochorismatase	SA
Solyc03g042560	157.66/74.40	73.37/108.51	1.08/−0.56	Phenylalanine ammonia-lyase	SA
Solyc05g026500	192.30/133.32	9.40/117.59	0.52/−3.64	Phenylalanine ammonia-lyase	SA
Solyc03g036470	151.84/103.25	—	0.55	Phenylalanine ammonia-lyase	SA
Solyc03g007070	1039.70/546.89	86.91/231.66	0.93/−1.41	1-aminocyclopropane-1-carboxylate synthase	ETH
Solyc08g079750	4118.98/3204.55	713.15/1285.27	0.36/−0.85	1-aminocyclopropane-1-carboxylate synthase	ETH

OE, SlDREBA4-overexpressed plants; EV, empty vector-transformed plants; VIGS: SlDREBA4-silenced plants; V1+V2, plants co-transformed by empty vectors pTRV1 and pTRV2; “—”, not detected.

**Figure 6:**
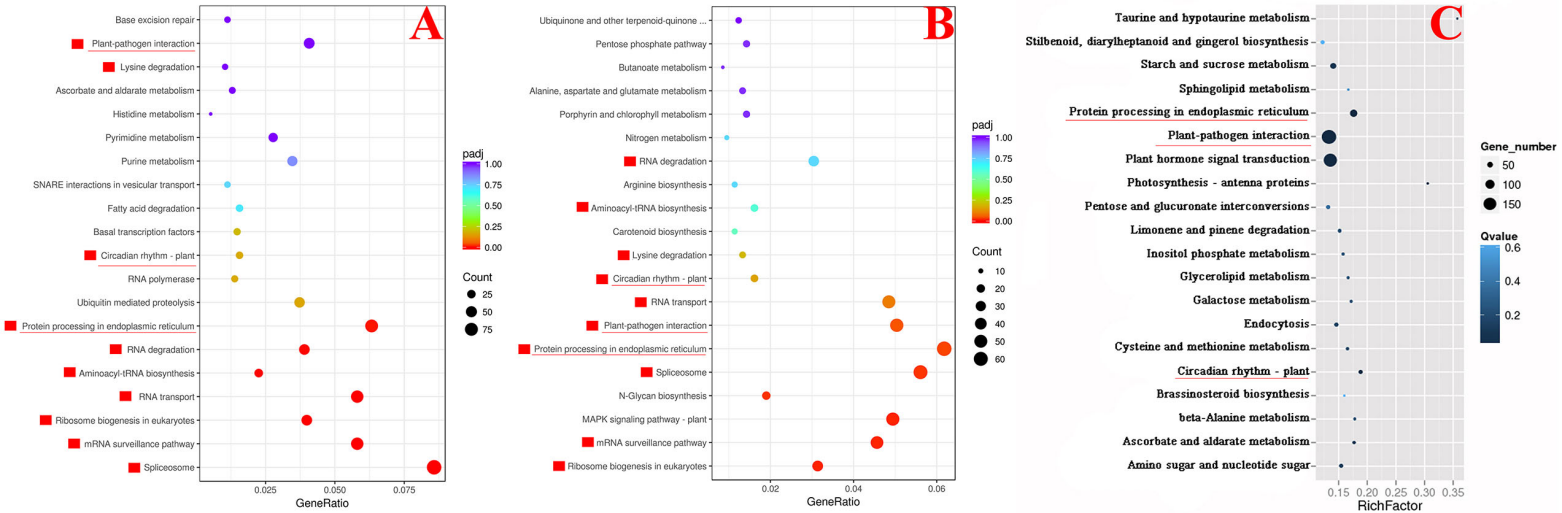
The KEGG dots representing the 20 most enriched pathway terms. **(A)** Genes up-regulated in overexpressed lines, **(B)** Genes down-regulated in silenced lines, **(C)** Genes differentially-expressed in high-temperature-induced Microtom plants. The red boxes represent the common pathway terms shared by **(A, B)**. Underlines indicate the common terms in **(A–C)**.

**Figure 7:**
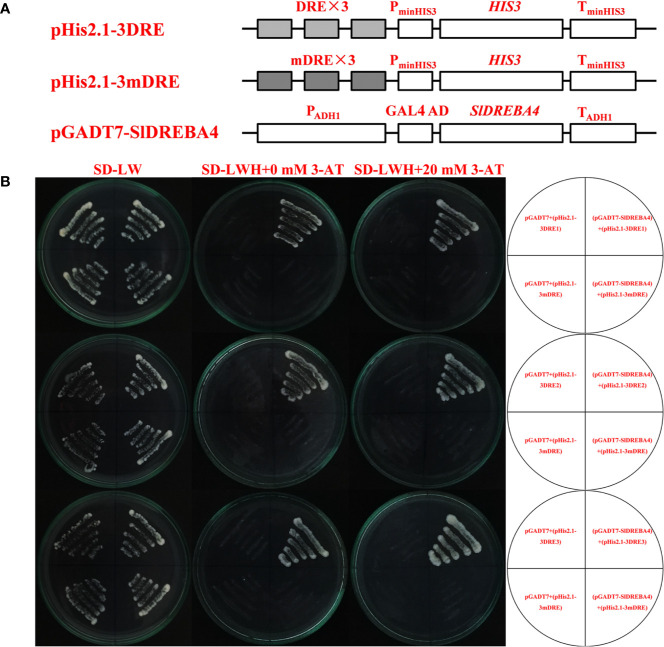
Analysis of DRE binding specificity of SlDREBA4. **(A)** Construction of the required vectors for the Y1H assay. The bait plasmid pHis2.1 contained three tandem repeat DREs or mDREs and the HIS reporter gene. The coding sequence of *SlDREBA4* was cloned into the activation domain GAL4 of the prey plasmid pGADT7. **(B)** Yeast strain Y187 was co-transformed with bait (pHis2.1-3DRE or pHis2.1-3mDRE) and prey (pGADT7 or pGADT7-*SlDREBA4*) construct. Interaction between the bait and prey was examined by cell growth on SD medium lacking Trp, Leu, and His (SD-WLH) containing 3-AT.

We compared the transcriptome results of *SlDREBA4*-overexpressed and -silenced lines with those of the empty vector-transformed plants to explain the changes in the tolerance of *SlDREBA4* transgenic plants to heat stress. There were 6333 (3978) DEGs in overexpressed (silenced) lines, including 2082 (1916) up- and 4251 (2062) down-regulated genes. An analysis of the KEGG enrichment pathways showed that 10 of the 20 pathways most enriched with up-regulated DEGs in *SlDREBA4*-overexpression lines were the same as those most enriched with down-regulated DEGs in VIGS lines (**Figures 6A, B**). Furthermore, three of the 20 most enriched pathways in the high-temperature-induced Microtom plants were shared by the above 10 common pathways (**Figure 6C**). One pathway was related to the circadian rhythms of plant (ko04712: circadian rhythms-plant) and the other two pathways were related to response to biotic stress and protein processing (ko04626: plant-pathogen interaction and ko04141: protein processing in endoplasmic reticulum). The common DEGs in the pathways of plant-pathogen interaction and protein processing in endoplasmic reticulum were discovered in the high-temperature-induced Microtom plants (up-regulated), S*lDREBA4*-overexpressed (up-regulated) and -silenced (down-regulated) lines. Two target genes which were described as heat shock proteins (Hsps) and calcium-binding protein were found in the pathway of plant-pathogen interaction. Seven target genes were found in the pathway of protein processing in endoplasmic reticulum, among which six were Hsps and one was skp1-like protein (**Table 4**). Further analysis of the DREs in the promoter sequences of the above 8 candidate genes found three potentially DREs (DRE1 [GACCGACGA], DRE2 [AGCCGACAC], DRE3 [CGCCGACTT]) which were located in the promoters of two *Hsp* genes (Solyc06g076570 and Solyc09g015020). The construction of the required vectors for the Y1H assay was shown in **Figure 7A**. Y1H experiment showed that yeast cells co-transformed with pGADT7-*SlDREBA4* and pHis2.1-3DRE grew well on the SD-LWH plates and even with 20 mM 3-amino-1,2,4-triazole (3-AT). However, the transformant yeast cells harboring the pGADT7-*SlDREBA4* and pHis2.1-3mDRE could not grow on SD-LWH plates (**Figure 7B**). The result indicated that SlDREBA4 possessed the ability to bind DRE as a general AP2 transcription factor.

**Table 4:** Common DEGs in the high-temperature-induced Microtom plants (up-regulated), *SlDREBA4*-overexpressed (up-regulated), and -silenced (down-regulated) lines.

Gene ID	Pathway	log2FoldChange	Definition
Solyc06g036290	Plant-pathogen interaction	8.47/1.82/−0.20	Hsp90 protein
Solyc10g079420	Plant-pathogen interaction	1.16/0.20/−0.39	Calcium-binding protein
Solyc08g062450	Protein processing in endoplasmic reticulum	9.74/3.25/−0.43	Class II small heat shock protein
Solyc06g076570	Protein processing in endoplasmic reticulum	7.41/1.09/−0.26	Class I small heat shock protein
Solyc09g015020	Protein processing in endoplasmic reticulum	7.38/1.16/−0.39	Class I heat shock protein
Solyc09g015000	Protein processing in endoplasmic reticulum	7.19/1.56/−0.45	Class I heat shock protein
Solyc04g014480	Protein processing in endoplasmic reticulum	6.78/2.12/−0.59	Heat-shock protein
Solyc11g008420	Protein processing in endoplasmic reticulum	3.98/0.74/−0.59	SKP1-like protein

## Discussion

DREB transcription factors are often differentially expressed in most high-temperature-induced transcriptomes (Wang et al., 2018; Xu and Huang, 2018). The DREBA4 subfamily genes, *AtTINY* (Wilson et al., 1996), *ZmDBF2* (Wang et al., 2010), *DaCBF4* (Byun et al., 2018), and *ZmDREB4.1* (Li et al., 2018) have been isolated. However, the response patterns and functions of these transcription factors under heat-stress conditions have not been well investigated. SlDREBA4 was the only DREBA4 subfamily transcription factor in the high-temperature-induced DGE profile of Microtom, and the transcription of *SlDREBA4* was induced by ABA, heat, drought, cold, and high salt. In addition to improving the resistance of transgenic plants to heat stress, SlDREBA4 could also increase resistance to high salt, drought, and cold (data not shown). The diversified stress responses and functions of SlDREBA4 provide new data and regulatory gene resources for future studies on molecular mechanisms of stress useful for tomato breeding.

Physiological systems in plants respond appropriately to avoid heat injury. Under heat-stress conditions, the levels of osmotic substances, including proline (Xu and Huang, 2018), soluble sugar and starch (Wang et al., 2018) increased, as did the contents of SA and ETH (Biddington and Robinson, 1993), JA (Xu and Huang, 2018) and other stress-related hormones. The antioxidant enzyme activities, including POD, SOD and CAT increased (Wang et al., 2018). AtDREB1C enhanced resistance to heat by increasing the activities of POD, SOD and CAT in transgenic lines (Wei et al., 2017). Under normal conditions, the contents of osmolytes and stress hormones, and the activities of antioxidant enzymes in *SlDREBA4*-overexpressed plants were greater than those in the empty vector-transformed lines. These results indicated that *SlDREBA4* regulated the thermotolerance through comprehensively altering the content of osmolytes and stress hormones, and the activities of antioxidant enzymes at the physiologic level.

A large number of protective proteins, such as Hsps, were synthesized under heat-stress conditions (Muthusamyab et al., 2017; Wang et al., 2018). Ca^2+^, as a central regulator of physiological responses to stress, plays an important role in regulating resistance to various stresses (Gong et al., 1998; Dodd et al., 2010; Wang et al., 2018). AtDREB2C and MsDREB2C increased the heat tolerance of overexpressed *Arabidopsis* by increasing the expression of *AtHsfA3* (Chen et al., 2010; Zhao et al., 2013). Six Hsps enriched in the protein processing in endoplasmic reticulum pathway and one calcium-binding protein enriched in the plant-pathogen interaction pathway were up-regulated in high-temperature-induced Microtom plants and *SlDREBA4*-overexpression lines, and were down-regulated in *SlDREBA4*-silenced lines. Here, three potentially DREs were identified in the promoters of two *Hsp* genes included in the above six *Hsps*, and bound with SlDREBA4, which demonstrated that SlDREBA4 transcription factor enhanced resistance to heat stress mainly through interactions with the *Hsps*.

Phytohormones are involved in plant growth and development, especially in adverse conditions. ABA plays a key role in plant acclimation to abiotic stress, including drought, heat, salt, and cold stress (Xiong et al., 2002; Suzuki et al., 2016; Zandalinas et al., 2016), and SA, JA, and ETH play regulatory roles in biotic stress (Dong et al., 1998). DREB transcription factors enhanced the resistance of transgenic plants to abiotic and biotic stress by increasing ABA, SA, JA, and ETH levels (Tsutsui et al., 2009; Zhao et al., 2013; Wu et al., 2017; Xiong et al., 2018). In our study, there was no significant difference in the ABA content in the empty vector-transformed with *SlDREBA4*-silenced and -overexpressed plants under normal growth and heat conditions. However, the SA, JA, and ACC contents in the empty vector-transformed plants were significantly higher than those in the *SlDREBA4*-silenced plants and lower than those in *SlDREBA4*-overexpressed lines. The KEGG pathway of plant-pathogen interaction (ko04626) included in the 20 most enriched pathway terms was obtained in the high-temperature-induced plants and *SlDREBA4*-overexpressed and -silenced lines. The above research result also indicates that SlDREBA4 plays a potential role in biotic stress responses, but the specific biotic stress in which SlDREBA4 functioned remains to be further explored.

## Conclusion

SlDREBA4 was determined to be a transcription factor of the DREBA4 family in tomato and plays an important regulatory role under heat-stress conditions. SlDREBA4 transcription factor enhances resistance to heat stress through interactions with the contents of osmolytes and stress hormones, and the activities of antioxidant enzymes at the physiological level and the expression of Hsps and calcium-binding protein at the molecular level.

## Data Availability Statement

All of the RNA-Seq datasets generated in this study are available from the SRA-Archive (http://www.ncbi.nlm.nih.gov/sra) under the study accession numbers: PRJNA554738, PRJNA579195, and PRJNA579731.

## Author Contributions

KZ designed the experiments and wrote the manuscript. LM, MD, and SJ generated the transgenic materials and performed the experiments. HZ analyzed the RNA-Seq data and provided assistance on vector construction. ZY measured the physiologic index. YY conducted the heat phenotyping experiment. All authors contributed to the article and approved the submitted version.

## Funding

This research was supported by the National Natural Science Foundation of China (31660576, 31760583), the Joint Project of Basic Agricultural Research in Yunnan Province (2018FG001-004), and the General Project of Yunnan Science and Technology plan (2016FB064).

## Conflict of Interest

The authors declare that the research was conducted in the absence of any commercial or financial relationships that could be construed as a potential conflict of interest.
