# Supplementary material for: Characterization of the DREBA4-Type Transcription Factor (SlDREBA4), Which Contributes to Heat Tolerance in Tomatoes
Source: Front Plant Sci. 2020 Sep 30;11:554520. doi: 10.3389/fpls.2020.554520 (PMC7554514; doi:10.3389/fpls.2020.554520)
Supplement: Supplementary file 1 [file Table_5.doc]

Table S1 Primers used for gene cloning and RT-qPCR

| Primer names | Forward (5'→3') | Reverse (5'→3') |
| --- | --- | --- |
| pSlDREBA4 | ATGTCAAAGCGAATAAGAGAGAGTG | TTATTTCATCATTTCAAAGTTGCTA |
| pSlDREBA4-q | TTGAGTCGGAAGAATCGAAGA | ATACCCATCAAAGTCGCCATC |
| pRPL2-q | CAGCGGATGTCGTGCTATGAT | GGGATGCTCCACTGGATTCA |
| pSlDREBA4-o | TCTAGAATGTCAAAGCGAATAAGAGAGAGTG (*Xba*I site underlined) | GGTACCTTTCATCATTTCAAAGTTGCTA (*Kpn*I site underlined) |
| pSlDREBA4-v | GAATTCACCGCGTAAAAAGTCACGC (*EcoR*I site underlined) | GGATCCTCCAGCTTGGAAGCTCTATAATT (*BamH*I site underlined) |
| Solyc01g009680-q | TAGTGATGGTTTAGTTTGGGAC | AAATAGCCTGCATAGCTGTATT |
| Solyc01g007920-q | TTCTACCAGCAATCAAAACCAC | CTTTATCCCCATTTCCAACAGT |
| Solyc03g042560-q | CCATTGATCGGTGTTTCAAGAA | GCAAAGAGCCACGAGATAGGTT |
| Solyc03g007070-q | GAATATTGGAAGTGGCGGTGAT | CCTACAGGGTTAGACGGGTTTG |
| Solyc08g079750-q | TTCACCAACCAGAACAACAACC | AACCTAAGAAAAGACCACCCCG |
| Solyc08g029000-q | CTGCCTTTGACGTTACGTTTG | GTTGGCTTTCTGCCTGTTCTT |
| Solyc04g039930-q | AACACACATTTTCCATTTCTTCC | TGCATTCCTAATTGATTTTAGGC |
| Solyc03g036470-q | TTTCTGCGTGGCTGGTATTAG | CGATCTTGCTTTCGTTTTTGA |
| Solyc01g007920-q | TTCTACCAGCAATCAAAACCAC | CTTTATCCCCATTTCCAACAGT |
| pSlDREBA4-y | GGATCCATGTCAAAGCGAATAAGAGAGAGTG (*BamH*I site underlined) | CCTCGAGGTTATTTCATCATTTCAAAGTTGCTA (*Xho*I site underlined) |
